# Unhealthy Food and Psychological Stress: The Association between Ultra-Processed Food Consumption and Perceived Stress in Working-Class Young Adults

**DOI:** 10.3390/ijerph18083863

**Published:** 2021-04-07

**Authors:** Matheus Lopes Cortes, José Andrade Louzado, Marcio Galvão Oliveira, Vanessa Moraes Bezerra, Sóstenes Mistro, Danielle Souto Medeiros, Daniela Arruda Soares, Kelle Oliveira Silva, Clávdia Nicolaevna Kochergin, Vivian Carla Honorato dos Santos de Carvalho, Welma Wildes Amorim, Sotero Serrate Mengue

**Affiliations:** 1 Multidisciplinary Health Institute, Federal University of Bahia, Rua Hormindo Barros, 58, Quadra 17, Lote 58, Bairro Candeias, Vitória da Conquista, 45029-094 Bahia, Brazil; louzado1@hotmail.com (J.A.L.); mgalvao@ufba.br (M.G.O.); vanessaenut@gmail.com (V.M.B.); smistro@gmail.com (S.M.); daniellesoutomedeiros@gmail.com (D.S.M.); danielaasoaresalves@gmail.com (D.A.S.); kelle.oliveira@gmail.com (K.O.S.); clavdian@yahoo.com.br (C.N.K.); vihonorato@hotmail.com (V.C.H.d.S.d.C.); 2 Department of Natural Sciences, State University of Southwest of Bahia, Estrada Bem Querer, Km-04, 3293, Bairro Candeias, Vitória da Conquista, 45083-900 Bahia, Brazil; welmawildes@hotmail.com; 3 Post-Graduate Program in Epidemiology, School of Medicine, Federal University of Rio Grande do Sul, Rua Ramiro Barcelos, 2400, Bairro Santa Cecilia, 90035-002 Porto Alegre, Rio Grande do Sul, Brazil; sotero@ufrgs.br

**Keywords:** stress, psychological stress, ultra-processed foods, eating, eating behavior

## Abstract

Background: Ultra-processed foods are industrial formulations made from food extracts or constituents with little or no intact food and often containing additives that confer hyper-palatability. The consumption of these products increases the risk of chronic non-communicable diseases. Stressed people may engage in unhealthy eating as a way to cope. This study aimed to verify whether ultra-processed food consumption was associated with perceived stress levels in industrial and retail workers from Vitoria da Conquista, Brazil. Methods: This was a cross-sectional study carried out between July 2017 and August 2018. During the study period, 1270 participants completed a survey administered by an interviewer. Stress levels were assessed using the Perceived Stress Scale. Information regarding weekly ultra-processed food consumption was collected. Ultra-processed foods were classified into four groups: sugary drinks; sugary foods; fast foods; and canned foods, frozen foods, or processed meat. The Student’s *t*-test or one-way analysis of variance was used to assess the differences in stress levels and ultra-processed food consumption. Ordinal regression was used to determine the association between the degrees of stress and ultra-processed food consumption levels. Results: Factors such as a young age, being unmarried, smoking, high-risk alcohol consumption, negative health perception, and high perceived stress level indicated higher rates of ultra-processed food consumption. Ordinal regression analysis showed that high stress levels were associated with increased odds of higher ultra-processed food consumption (odds ratio: 1.94; 95% CI: 1.54–2.45). Conclusions: These findings could help identify appropriate target areas for interventions aimed at mental health promotion and healthier food consumption.

## 1. Introduction

Ultra-processed foods are made from food extracts or constituents such as fats, starches, and added sugars. They contain little or no intact food but include flavorings, colorants, and other additives that confer hyper-palatability to humans. They are typically ready to eat or can be heated up quickly; are energy-dense, high glycemic, fatty, or salty; and are poor in dietary fiber, protein, various micronutrients, and other bioactive compounds. Examples include sweet, fatty, or salty packaged snack products; ice cream; sugar-sweetened beverages; chocolates; confectioneries, French fries; burgers; hot dogs; and poultry and fish nuggets [1,2].

Consuming high amounts of ultra-processed foods makes energy regulation difficult, which increases the risk of weight gain and chronic non-communicable diseases (NCDs) such as cardiovascular diseases, type 2 diabetes, and cancers [3,4].

Several factors might affect diet patterns/quality, such as adiposity, smoking, age, income, educational level, and marital status. However, food consumption patterns and their relationship with psychological conditions, such as stress, are receiving more research attention at present [5,6]. Psychological stress occurs when an individual perceives that environmental demands tax or exceed his or her adaptive capacity [7].

Stressed people may engage in health-risk behaviors such as unhealthy eating, smoking, and drinking alcohol. Risky—but often pleasurable—behaviors are ways to cope with stress [8,9,10,11,12,13]. A recent study conducted with Brazilian workers showed that high levels of perceived stress were associated with smoking, obesity, and the co-occurrence of health-risk behaviors [14]. Chronic stress is positively associated with highly palatable, nutrient-poor food intake. The consumption of highly palatable foods—such as those high in fat, sugar, or salt—may activate the endogenous opioid (reward) system and reduce the hypothalamic-pituitary-adrenal (HPA) axis stress response, thereby alleviating stress symptoms. Additionally, highly palatable food intake may reduce stress via sensory pleasure, distraction, escape, or other nutritional or metabolic effects [15,16].

Most studies examining the relationship between perceived stress and eating behaviors were conducted among college students. In these individuals, higher stress was associated with a greater preference for ultra-processed foods, such as sweet foods, commercially pre-prepared or pre-packaged mixed dishes (a mixed dish generally includes foods from two or more food groups—e.g., casseroles, frozen entrees/dinners, stews, pizza, or lasagna); increased snack-type food consumption; and a decreased consumption of meal-type foods such as fruits, vegetables, meat, and fish [17]. However, little is known about the relationship between stress and diet in young adults, such as workers. Understanding stress–diet relationships in this population may be particularly important because they are disproportionately exposed to stressful work conditions.

In Brazil, workers face great difficulties in accessing health services. Basic health facilities are available only during the day, and workers are unable to leave the workplace and use these services. As a result, there is a huge loss of opportunities for stress diagnosis and treatment in this population and, consequently, a higher likelihood of health-risk behaviors. This contributes to a reduced quality of life, high absenteeism rates, high staff turnover, reduced productivity, reduced ability to perform work safely, increased costs, and reduced employer profits. Therefore, this study aimed to verify whether ultra-processed food consumption was associated with the perceived stress levels in industrial and retail workers in Vitoria da Conquista, Brazil.

## 2. Materials and Methods

### 2.1. Design

This study employed a cross-sectional design. It was a part of the HealthRise global initiative’s longitudinal HealthRise Vitória da Conquista project. The HealthRise initiative was developed to implement and evaluate pilot programs aimed at improving the screening, diagnosis, management, and control of non-communicable diseases among underserved communities [18].

### 2.2. Data Collection

An anonymous questionnaire was prepared based on the instrument used in the Brazilian National Health Survey [19] and consisted of five categories: household, sociodemographic characteristics, habits and behaviors, health/illness, and objective measures. The Brazilian National Health Survey collected information on the performance of the national health system concerning access to and use of available services and care continuity. It also collected data on the health condition of the population, the surveillance of chronic non-communicable diseases, and the associated risk factors. Face-to-face interviews were conducted from July 2017 to August 2018.

### 2.3. Participants

A representative sample of workers from the Industry Social Service in Vitória da Conquista was selected. The Industry Social Service is a private nationwide institution that supports industries and workers with health education and promotion activities, health care, rehabilitation, and common disease diagnosis. Participants were recruited while they waited for a medical appointment with an occupational physician and offered an option to participate or not. The inclusion criteria were age ≥18 years, living in the municipality, and having made periodic appointments with an occupational physician. The exclusion criteria were workers from other municipalities or those awaiting a dismissal medical evaluation. During the study period, 3727 workers scheduled appointments with an occupational physician. However, 339 did not show up, 833 were awaiting a dismissal medical evaluation, 516 were from other municipalities, 25 were under 18 years, and 744 refused to participate. Finally, 1270 individuals were included for analysis.

### 2.4. Measures

#### 2.4.1. Demographic and Socioeconomic Characteristics

Participants were asked to provide their basic demographic information including sex, age, educational and socioeconomic levels, and marital status. The socioeconomic level was categorized according to the Brazil Economic Evaluation Criteria [20]. These criteria evaluate individuals’ socioeconomic levels based on a household assessment. Scores range from 0 to 100 points, with higher scores representing belonging to a higher economic stratum: A (45–100 points), B1 (38–44 points), B2 (29–37 points), C1 (23–28 points), C2 (17–22 points), and D/E (≤16 points).

#### 2.4.2. Ultra-Processed Food Consumption

Information regarding the participants’ weekly ultra-processed food consumption was collected. Ultra-processed foods were classified into four groups: sugary drinks (soda, soft drinks); sugary foods (biscuits, cookies, cakes, pie, chocolates, candies); fast foods (hamburgers, pizza); and canned foods, frozen foods, or processed meat (salami, ham, sausage, bacon). The introductory question, ‘How many days of the week do you eat/drink…?’ asked participants about their weekly consumption frequency of each food group separately. The responses ranged from 0 to 7 days a week. Ultra-processed food consumption in the past week was converted into a scale of 0 to 28 points by multiplying the number of ultra-processed food groups consumed by the number of days consumed, where 0 indicates no consumption over the past week and 28 indicates all groups were consumed every day. Ultra-processed food consumption was categorized into tertiles and classified as follows: low consumption (≤5 points), moderate consumption (6–9 points), and high consumption (≥10 points).

#### 2.4.3. Perceived Stress

The 10-item Perceived Stress Scale (PSS) [21] is one of the most widely used instruments for perceptions of stress measurement. In this study, we used the version translated and validated for use with Brazilian Portuguese populations [22,23,24]. Perceived stress levels were categorized into tertiles and classified as follows: low (≤12 points), moderate (13–18 points), and high (≥18 points).

#### 2.4.4. Health Indicators

We collected data on body mass index (BMI) status and self-rated health (SRH). Weight and height were measured, and BMI was calculated. With the respondents dressed in light clothing, weight was measured with an electronic scale accurate to within 0.1 kg. Height was measured using a portable stadiometer accurate to within 0.1 cm while the respondents stood barefoot and looked at a fixed point at eye level. Participants were classified into two categories: BMI ≤ 24.9 kg/m^2^ and BMI ≥ 25 kg/m^2^ [25].

SRH was used as a general health status measure based on the following item: ‘In general, how would you rate your health?’ Responses included: ‘very good’, ‘good’, ‘neither good nor poor’, ‘poor’, and ‘very poor’. Based on the responses, SRH was dichotomized into good (very good/good) and poor (neither good nor poor/poor/very poor).

#### 2.4.5. Behavioral Variables

Behavioral variables were assessed using the following indicators: smoking, alcohol consumption, and physical activity. Participants were considered smokers if they used any tobacco products, regardless of the amount or periodicity. Alcohol consumption was considered high-risk when the intake on the same occasion in the last 30 days was four or more drinks for women and five or more drinks for men [19]. Physical activity—assessed using the short version of the International Physical Activity Questionnaire and translated and validated for use with Brazilian Portuguese populations [26]—was measured by multiplying the weekly frequency (days) by the average duration (minutes) of moderate or vigorous activity. The time spent on vigorous activity was multiplied by two. Only physical activity performed for at least 10 continuous minutes was validated. Workers who engaged in less than 150 min of physical activity per week were considered inactive [27,28].

### 2.5. Statistical Analysis

Analyses were conducted using SPSS Statistics version 27 (IBM Corp., Armonk, NY, USA). Descriptive data were presented as means and standard deviation (*SD*) for continuous variables and frequencies and percentages for categorical variables. The normality of the data was tested by the Shapiro–Wilk test. To compare the ultra-processed food consumption between characteristics and the perceived stress level by the weekly frequency of ultra-processed food consumption, the Student’s *t*-test (variables with two categories) or one-way analysis of variance (variables with three or more categories) was used, with the application of Tukey’s honestly significant difference test (variable ‘Perceived Stress Level’) or the Kruskal–Wallis test (the variables were ‘Age’ and ‘Educational Level’) owing to homogeneity or non-homogeneity of variances, respectively.

A cumulative odds ordinal logistic regression with proportional odds was run to determine the perceived stress levels’ effect (independent variable) on ultra-processed foods consumption (dependent variable). The predictors were tested a priori to verify there was no multicollinearity. Unadjusted and adjusted odds ratios (ORs) for demographic, socioeconomic, behavioral, and health indicator variables were determined. The proportional odds assumption was assessed by a full likelihood ratio test comparing the fitted model to a model with varying location parameters. The deviance goodness-of-fit test indicated that the model was a good fit to the observed data, and the final model statistically and significantly predicted the dependent variable. The estimates were calculated using points and 95% confidence intervals (CIs). The models were analyzed with the complete data table. There was no imputation of missing data.

### 2.6. Ethical Considerations

All the participants provided written informed consent. The study was approved by the Ethics Committee of the Multidisciplinary Health Institute, Federal University of Bahia (62259116.0.0000.5556).

## 3. Results

Demographic, socioeconomic, and behavioral characteristics, as well as educational level, BMI status, SRH, perceived stress level, and ultra-processed food consumption in the sample, are presented in Table 1. Most respondents were men, who had a mean age of 33 years (*SD* = 9.8) and were married. The most prevalent educational level was any high school or professional training. Most respondents belonged to the C1 and C2 socioeconomic classes. Regarding behavioral variables, most workers exhibited good lifestyle habits. An important proportion (35.8%) of participants had a negative health perception. Young workers had a significantly higher ultra-processed food consumption. Additionally, ultra-processed food consumption was higher in unmarried workers, smokers, those with high-risk alcohol consumption, with negative SRH, and with a high perceived stress level. On the opposite end, workers with no education or up to an elementary-level education had lower ultra-processed food consumption compared to those with higher education levels. The normality test showed that the measured variables had a normal distribution.

The workers who consumed ultra-processed foods at least once a week had significantly higher perceived stress levels compared to those who did not consume any, regardless of food type. Additionally, the perceived stress levels were higher in workers who consumed more ultra-processed food groups, as shown in Table 2.

Table 3 shows the associations between stress and ultra-processed food consumption. Workers with high perceived stress had 83% higher odds (OR: 1.83; 95% CI: 1.47. 2.27) of higher ultra-processed food consumption compared to the moderate- and low-stress groups. When the model was adjusted for demographic, socioeconomic, and behavioral variables; BMI status; and SRH variables, the odds remained 1.94 (95% CI: 1.54. 2.45) times higher.

## 4. Discussion

Although the consumption of unhealthy foods appears to be greater in stressed individuals, there is a gap in knowledge in this area for young adult workers. In this study, we investigated the association between ultra-processed food consumption and the perceived stress levels in workers. We found significantly higher ultra-processed food consumption among young workers (18–35 years), those with a higher educational level, who were unmarried, were smokers, had high-risk alcohol consumption, had negative SRH, and high perceived stress levels. Additionally, we found that workers who consumed ultra-processed foods had significantly higher perceived stress levels. Moreover, higher perceived stress levels were associated with increased odds of higher ultra-processed food consumption.

Stress has direct and indirect adverse effects on health, and one way stress may affect health is by influencing the foods people choose to eat. Stress appears to increase food consumption in certain individuals and is a significant instigator of poor eating behaviors, especially in the young adult population [29,30]. 

Various kinds of psychological stressors are implicated in overeating and poor eating choices. Prolonged occupational stress is associated with higher energy consumption, saturated fat, and sugar intake, and possible weight gain, especially in restrained eaters or those who intentionally restrict their consumption. Stress-induced social situations also increase the consumption of highly palatable foods. Greater perceived stress in both men and women is associated with a higher fat diet and less frequent exercise. A study with low-income pregnant women showed that women with obesity and women who experience a higher level of psychosocial stress or more depression are more likely to eat fast foods than their counterparts [31,32].

Research in humans has focused on major stress from irritations and aggravations, which were associated with an increased consumption of snacks high in fat or sugar. Moreover, studies have found relationships between negative emotions (e.g., depressive symptoms) or emotional eating (tendency to eat in response to negative emotions) and poorer dietary behaviors. Chronic stress was also positively associated with highly palatable, nutrient-poor food intake (e.g., chips, fried foods, burgers, sweetened beverages). Highly palatable foods induce physical reactions that reduce stress. Some activate the endogenous opioid (reward) system and reduce the hypothalamic-pituitary-adrenal (HPA) axis stress response. Highly palatable food intake may also reduce stress via sensory pleasure, distraction, or escape, as well as other nutritional or metabolic effects [16].

Studies on stress-related dietary changes have focused on eating behaviors such as emotional eating, external eating, and restrained eating as enhancers of stress-induced eating [10]. Within the domain of emotional eating, two models of thought exist: the General Effects and Individual Differences models. The former has been examined predominantly in animals and the latter exclusively in humans. According to the General Effects Model, all organisms will increase food intake in response to stress. The Individual Differences Model posits that certain factors of the individual will dictate whether or not stress leads to eating. One of the main hypotheses of the Individual Differences Model that has been tested is that obese individuals are more likely to engage in stress-induced eating than normal-weight individuals [33]. Other individual factors include food preference, the importance of health, activity level, and self-control. Self-control-related psychological factors such as impulsivity, reward sensitivity, and stress coping have been related to dietary intake [10].

The mechanism through which stress influences food choices involves hormonal interactions and metabolic processes, as well as individual differences in psychological and neurochemical responses to stress and eating. Stress is associated with reduced levels of insulin and leptin, which interact and influence changes in appetite. It elicits a more passive response driven by the hypothalamic-pituitary-adrenal, with an increase in cortisol that may entice people to consume hedonic, energy-dense foods and potentially lead to unwanted weight gain and obesity [34].

Perhaps the association between stress and food consumption goes in the opposite direction, with poor eating behaviors indicating a risk factor for high stress. In this regard, consumption affects the way humans feel. Research on the diets and lifestyles of volunteers who were not experiencing depression found that participants with a high consumption of trans-fats (pastries and fast food) had up to a 48% increase in risk of depression when compared to participants who did not consume these fats [29]. Ultra-processed food consumption increases the risk of excessive weight gain and obesity. People with obesity are more frequently dissatisfied with their bodies compared to those with a normal weight, affecting perceived stress [35]. Additionally, a study has shown a positive association between perceived stress and the combination of high unhealthy food consumption, such as junk food, and a negative association with healthy foods, such as fruits and vegetables [36]. Although reverse causality is possible, once stress development occurs, this mental health problem will lead to the adoption of health-risk behaviors, such as unhealthy eating.

Our study had some limitations. First, the cross-sectional design prevented us from distinguishing between causes and consequences. Second, we did not evaluate the surrounding food environment. Stress may be particularly likely to increase consumption of foods high in sugar, fat, and/or salt when these options are readily accessible in workplaces or local neighborhoods with more convenience stores and restaurants, especially fast-food restaurants. Third, the instrument used to collect food information was not designed to evaluate the extent of calories consumed that came from ultra-processed foods. Finally, we did not seek information about eating out.

One strength of this study was its large sample size. This was important as workers, especially young men, have a relatively low likelihood of participating in health research and tend to be underrepresented in health profile surveys. Furthermore, there is a scarcity of studies on perceived stress levels and ultra-processed food consumption in workers. In this study, we simultaneously examined the isolated consumption of four groups of ultra-processed foods, as well as the aggregate consumption of these groups, and assessed the consumption of ultra-processed foods with an easy method that can help healthcare professionals investigate their patients’ food choices and improve their habits.

## 5. Conclusions

Young, unmarried workers who were smokers, with high-risk alcohol consumption, negative SRH, and high perceived stress levels had higher levels of ultra-processed food consumption. Workers with no education or up to an elementary-level education had lower ultra-processed food consumption. High stress levels were associated with increased odds of higher ultra-processed food consumption. Longitudinal studies are needed to examine the causal association between perceived stress and ultra-processed food consumption, facilitating a better understanding of the pathways through which these variables are related. We found that poor nutritional habits are associated with stress symptoms in workers; therefore, interventions aimed at mental health promotion in these individuals may also lead to the consumption of healthier foods, and vice-versa.

## Figures and Tables

**Table 1:** The distribution of ultra-processed food consumption by demographic, socioeconomic, and behavioral variables; health indicators; and perceived stress level.

Variable	*N* (%)	Ultra-Processed Food Consumption (SD) ^a^	*p* ^b^
**Sex**			
Men	1019 (80.2)	7.5 (4.7)	0.07
Women	251 (19.8)	6.9 (4.7)	
**Age (years)**			
≤25	279 (22.0)	8.7 (5.1)	<0.05 *^d^
26–30	259 (20.4)	7.8 (4.6)	
31–35	265 (20.9)	7.5 (4.5)	
36–40	192 (15.1)	6.8 (4.7)	
≥41	275 (21.7)	5.9 (4.4)	
**Educational Level** ^c^			
Undergraduate or up to Postdoctoral-level	327 (25.8)	7.5 (4.6)	<0.05 *^e^
Any High School or Professional Training	634 (50.0)	7.8 (4.8)	
No education or up to Elementary-level education	306 (24.2)	6.4 (4.6)	
**Socioeconomic Status**			
A/B1/B2	465 (36.6)	7.5 (4.6)	0.17
C1/C2	629 (49.5)	7.5 (4.8)	
D/E	176 (13.9)	6.8 (4.5)	
**Marital Status** ^c^			
Married	789 (62.2)	7.2 (4.6)	0.04 *
Single/Divorced/Widowed	480 (37.8)	7.7 (5.0)	
**Smoking** ^c^			
No	107 (8.4)	7.3 (4.7)	0.02 *
Yes	1162 (91.6)	8.6 (5.5)	
**High-Risk Alcohol Consumption**			
No	364 (28.7)	7.1 (4.6)	<0.01 *
Yes	906 (71.3)	8.2 (4.9)	
**Physical Activity Status**			
Active	791 (62.3)	7.3 (4.8)	0.69
Inactive	479 (37.7)	7.5 (4.7)	
**BMI Status** ^c^			
BMI ≤ 24.9 kg/m^2^ BMI ≥ 25 kg/m^2^	647 (51.3) 614 (48.7)	7.6 (4.9) 7.1 (4.5)	0.08
**Self-Rated Health** ^c^			
Good	815 (64.2)	7.1 (4.6)	0.01 *
Poor	454 (35.8)	7.9 (5.0)	
**Perceived Stress Level** ^c^			
Low	435 (34.5)	6.5 (4.5)	<0.05 *
Moderate	395 (31.4)	7.1 (4.3)	
High	429 (34.1)	8.6 (5.1)	

^a^ Ultra-processed food consumption in the past week (number of consumed groups x days a week). ^b^ Student’s *t*-test and one-way analysis of variance. ^c^ Variables that not all participants responded to in the survey. * *p* < 0.05. *^d^ Tukey HSD Test: AxC, AxD, AxE; BxE; CxE. *^e^ Tukey HSD Test: AxB; AxC.

**Table 2:** Perceived stress by weekly frequency of ultra-processed food consumption in workers.

Variable	*N* (%)	Perceived Stress Level (*SD*)	*p* ^a^
**Sugary drinks**			
No Consumption	406 (32.0)	14.4 (6.1)	0.049 *
Consumption	864 (68.0)	15.1 (6.1)	
**Sugary foods**			
No Consumption	221(17.4)	13.6 (6.6)	<0.01 *
Consumption	1049 (82.6)	15.1 (6.0)	
**Fast foods**			
No Consumption	768 (60.5)	14.1 (6.2)	<0.01 *
Consumption	502 (39.5)	16.1 (5.7)	
**Canned foods, frozen foods or processed meat**			
No Consumption	414 (32.6)	14.0 (6.4)	<0.01 *
Consumption	856 (67.4)	15.3 (5.9)	
**Aggregate consumption (two categories)**			
No group consumed	47 (3.7)	12.5 (6.8)	0.01 *
One or more groups consumed	1223 (96.3)	15.0 (6.1)	
**Aggregate consumption (five categories)**			
No group consumed	47 (3.7)	12.5 (6.8) A	
One group consumed	184 (14.5)	13.4 (6.4) B	
Two groups consumed	319 (25.1)	14.7 (6.4) C	<0.05 *^b^
Three groups consumed	431 (33.9)	14.8 (5.6) D	
Four groups consumed	289 (22.8)	16.5 (5.8) E	

Note. ^a^ One-way analysis of variance. * *p* < 0.05. *^b^ Tukey HSD Test: AxE, BxE, CxE; DxE.

**Table 3:** Associations between ultra-processed food consumption and perceived stress levels in workers.

	Unadjusted	Adjusted				
	OR 95% CI	OR ^a^ 95% CI	OR ^b^ 95% CI	OR ^c^ 95% CI	OR ^d^ 95% CI	OR ^e^ 95% CI
**Perceived Stress Level**						
Low/Moderate	1.0	1.0	1.0	1.0	1.0	1.0
High	1.83 (1.47–2.27)	2.01 (1.60–2.51)	1.86 (1.48–2.33)	2.02 (1.61–2.55)	2.00 (1.59–2.52)	1.94 (1.54–2.45)
**Sex**						
Women		1.0	1.0	1.0	1.0	1.0
Men		1.49 (1.14–1.94)	1.76 (1.32–2.35)	1.49 (1.11–1.99)	1.49 (1.11–2.01)	1.53 (1.14–2.06)
**Age (years)**						
≥41		1.0	1.0	1.0	1.0	1.0
36–40		1.32 (0.93–1.88)	1.47 (1.02–2.10)	1.26 (0.88–1.81)	1.28 (0.89–1.84)	1.27 (0.89–1.83)
31–35		1.73 (1.25–2.40)	1.86 (1.34–2.59)	1.65 (1.19–2.30)	1.70 (1.22–2.38)	1.71 (1.23–2.39)
26–30		2.18 (1.58–3.02)	1.98 (1.41–2.78)	2.00 (1.43–2.81)	2.07 (1.46–2.92)	2.11 (1.49–2.98)
≤25		2.72 (1.97–3.74)	3.27 (2.29–4.66)	2.88 (2.02–4.11)	3.00 (2.07–4.34)	3.01 (2.08–4.36)
**Educational Level**						
Undergraduate or up to Postdoctoral-level			1.0	1.0	1.0	1.0
Any High School/Professional Training			0.97 (0.73–1.29)	0.89 (0.65–1.18)	0.89 (0.67–1.19)	0.87 (0.66–1.17)
No education or up to Elementary-level education			0.68 (0.47–0.99)	0.59 (0.41–0.86)	0.59 (0.41–0.86)	0.59 (0.40–0.86)
**Socioeconomic Status**						
A/B1/B2			1.0	1.0	1.0	1.0
C1/C2			0.96 (0.74–1.23)	1.06 (0.82–1.36)	1.05 (0.81–1.35)	1.03 (0.80–1.33)
D/E			0.91 (0.62–1.32)	0.88 (0.60–1.29)	0.89 (0.61–1.30)	0.88 (0.60–1.29)
**Marital Status**						
Single/Divorced/Widowed			1.0	1.0	1.0	1.0
Married			1.21 (0.95–1.54)	1.24 (0.97–1.58)	1.23 (0.97–1.57)	1.22 (0.96–1.56)
**Smoking**						
No				1.0	1.0	1.0
Yes				1.80 (1.20–2.69)	1.79 (1.19–2.69)	1.76 (1.17–2.65)
**High-Risk Alcohol Consumption**						
No				1.0	1.0	1.0
Yes				1.37 (1.08–1.74)	1.36 (1.07–1.74)	1.37 (1.08–1.75)
**Physical Activity Status**						
Active				1.0	1.0	1.0
Inactive				1.12 (0.90–1.39)	1.12 (0.90–1.40)	1.11 (0.89–1.38)
**BMI Status**						
BMI ≤ 24.9 kg/m^2^					1.0	1.0
BMI ≥ 25 kg/m^2^					1.07 (0.85–1.34)	1.05 (0.84–1.32)
**Self-Rated Health**						
Good						1.0
Poor						1.24 (0.99–1.55)

^a^ Adjusted for sex and age. ^b^ Adjusted for sex, age, educational level, socioeconomic status, and marital status. ^c^ Adjusted for sex, age, educational level, socioeconomic status, marital status, smoking, high-risk alcohol consumption, and physical activity status. ^d^ Adjusted for sex, age, educational level, socioeconomic status, marital status, smoking, high-risk alcohol consumption, physical activity status, and BMI status. ^e^ Adjusted for sex, age, educational level, socioeconomic status, marital status, smoking, high-risk alcohol consumption, physical activity status, BMI status, and self-rated health. OR, odds ratio; CI, confidence interval.

## Data Availability

The data that support the findings of this study are available from the corresponding author, M.L.C., upon reasonable request.
